# SCALP and temporal fascia in the cranio-orbito-zygomatic approach: highlighting the interfascial dissection plane

**DOI:** 10.1007/s00701-026-06947-8

**Published:** 2026-06-10

**Authors:** Tufan Agah Kartum, Ayberk Karaman, Levent Aydın, Eren Soğuk, Revna Çetiner, Tuba Özge Karaçoban, Kardelen Akıncı, Necmettin Tanriover

**Affiliations:** 1 Department of Neurosurgery, Cerrahpasa Medical Faculty, Istanbul University-Cerrahpaşa, 34098 Fatih, Istanbul, Turkey; 2 Microsurgical Neuroanatomy Laboratory, Department of Neurosurgery, Cerrahpasa Medical Faculty, Istanbul University-Cerrahpaşa, Istanbul, Turkey

**Keywords:** Temporal fascia, Facial nerve, Orbitozygomatic craniotomy, SCALP, Temporoparietal fascia, Zygomatic arch

## Abstract

**Background:**

Cranio-orbito-zygomatic approach (COZA) requires adequate SCALP, fascia and muscle retraction to access the zygomatic arch, malar eminence (ME) and inferior orbital fissure (IOF), which endanger the facial nerve branches. Performing skull base reconstruction also demands a layer-by-layer understanding of the SCALP and fasciae. We aimed to examine the relationship between the SCALP layers, temporal fascia (TF) and facial nerve branches from a surgical viewpoint and highlight the optimal dissection plane during the COZA.

**Method:**

The SCALP and temporal fascia layers were examined using COZA on 10 formalin-fixed cadavers (20 sides). Stepwise dissections analyzed the relationships between SCALP layers, temporoparietal fascia, TF and facial nerve branches. In addition, facial dissections were conducted to examine the relationships between the facial nerve branches and the associated fasciae.

**Results:**

The temporoparietal fascia was continuous with the galea superomedial to superior temporal line. Facial nerve branches coursed between the galea and the TF. The TF divided into superficial and deep layers; superficial layer continuous with the pericranium and extending anteriorly toward periorbita. Following the superficial layer of the TF inferiorly provided a natural interfascial surgical plane for exposure of the ME. Deep layer of the TF further divided into medial and lateral laminae with an interfascial fat pad between them. The lateral and medial laminae attached to the lateral and medial surfaces of the zygomatic arch, respectively, and merged at the superior margin.

**Conclusıons:**

Recognizing the interfascial plane between the superficial and deep layers of the TF and the deep interfascial fat pad, may improve surgical orientation and facilitate tissue retraction during exposure of the lateral orbital wall and ME in the COZA.

## Introduction

Fundamental surgical skills encompass a comprehensive knowledge of microsurgical anatomy and the ability to safely expose the targeted area by accurately identifying the correct plane of dissection [9, 36]. Concept of the ‘tissue plane’ emphasizes the importance of ‘creating’ a plane of dissection by entering the correct anatomical plane, allowing safe exposure of the surgical field while preserving functional nerves [9, 10, 36]. This principle becomes particularly critical in cranio-orbito-zygomatic approach (COZA), especially during elevation of the SCALP layers and temporal fascia (TF).

COZA requires adequate SCALP and temporalis muscle retraction to access the zygomatic arch, malar eminence (ME) and inferior orbital fissure (IOF) prior to osteotomies, which endanger facial nerve branches [3, 4, 6, 15, 16, 21, 25, 32, 34, 39]. Frontotemporal skin incisions pose unique difficulties due to complex anatomical relationships between the SCALP layers, TF and relevant neurovascular structures. In addition, skull base reconstruction using galeal and pericranial flaps requires a precise understanding of continuity between these fascial layers [27]. Preservation of vascular supply and neural integrity is essential to prevent SCALP ischemia and temporalis muscle atrophy [24].

Interfascial and subfascial dissection techniques have been described to preserve facial nerve during pterional and orbitozygomatic craniotomies [11, 37]. Although these techniques are well established and widely practiced, variations in terminology and inconsistent descriptions of TF layering may create uncertainty during operative dissection. A clearer stepwise understanding of relationships between the SCALP layers, temporoparietal fascia (TPF), TF and facial nerve branches—particularly at level of the superior temporal line (STL) and zygomatic arch—may facilitate a more reproducible surgical strategy.

In this study, we examined the relationships between the SCALP layers, TF and facial nerve branches from a surgical perspective. We aimed to provide a detailed microsurgical analysis of these layers during COZA to highlight the optimal fascia–muscle dissection plane that may assist safe exposure of the ME and lateral orbital wall while maintaining facial nerve integrity.

## Material and methods

Ten formalin-fixed, silicone-injected cadaveric heads (20 sides) were examined to reveal relationships between layers of SCALP and TF and neurovascular structures by performing COZA. In each specimen, frontotemporal skin incisions were made and a stepwise dissection was performed to expose SCALP layers, TPF, TF, interfascial fat pad and their relationships to facial nerve branches, zygomatic arch, ME and STL. Facial dissections were also performed to examine the facial nerve branches.

Dissections were performed under a Zeiss Surgical Microscope (Carl Zeiss AG, Oberkochen, Germany) at × 6 to × 40 magnification. Three-dimensional images of each step of dissection were obtained.

## Results

### Stepwise frontotemporal skin incision based on the SCALP layer dissections

**Fig. 1:**
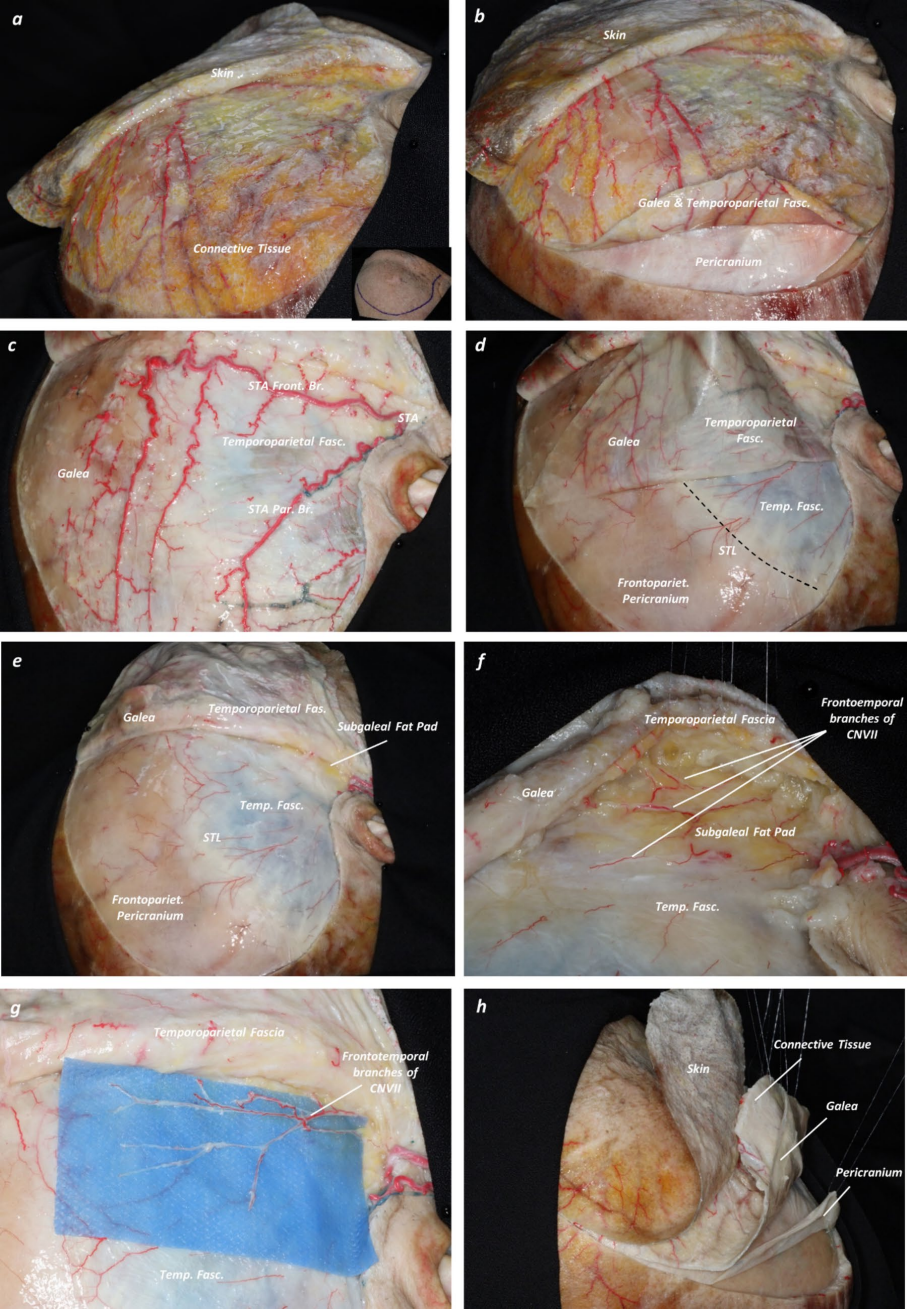

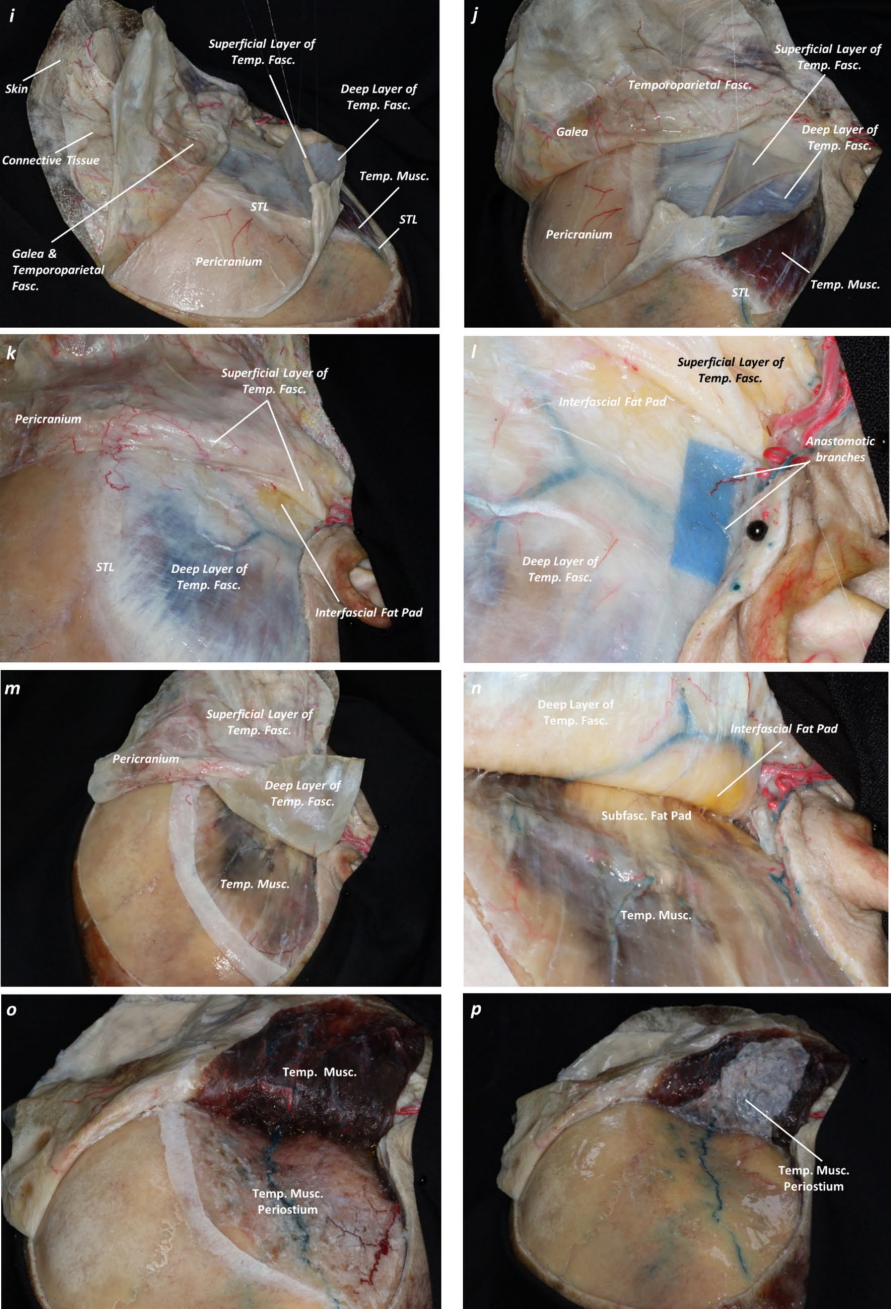
Layer by layer SCALP dissection following frontoparietotemporal skin incision. **a** After a right-sided frontoparietotemporal skin incision with the head rotated 45 degrees to the contralateral side, the “S” layer was removed and the connective tissue and galea layer were exposed. The distal thin branches of the STA are located within the connective tissue. *(The inset shows the skin incision*.*).* **b** The galea and temporoparietal fascia have been dissected together to expose the pericranium located deep to them. **c** After removing the connective tissue, the galea and temporoparietal fascia have been exposed. The galea continues as the temporoparietal fascia inferior and lateral to the superior temporal line (STL). The main trunk of the STA and the proximal frontal and parietal branches are located within the temporoparietal fascia. **d** By retracting the galea and temporoparietal fascia together anteriorly, the pericranium and temporal fascia have been revealed in a deeper layer. The pericranium continues as the temporal fascia inferior and lateral to the STL. The galea continues as the temporoparietal fascia inferior and lateral to the STL. *(Black dashed lines indicate STL.).* **e** Deep to the galea, the loose areolar tissue layer between the galea and the pericranium provides a good dissection plane between the galea and the pericranium. The galea and temporoparietal fascia have been retracted more anteriorly over the pericranium and temporal fascia, to disclose the subgaleal fat pad situated between the temporoparietal fascia and the temporal fascia. **f-g** The subgaleal fat pad has been displayed between the temporoparietal fascia and the temporal fascia within the loose areolar tissue. The frontotemporal branches of the facial nerve are located within the subgaleal fat pad. **h–j** The temporal fascia – continuous with the pericranium – has been separated into two layers, superficial and deep, at the level of the STL. The temporalis muscle is located deep to the deep layer of the temporal fascia and attaches to the STL. The subgaleal fat pad is located between the temporoparietal fascia and the superficial layer of the temporal fascia. **k** By retracting the superficial layer of the temporal fascia anteriorly, the reflexion of the interfascial fat pad located within the deep layer of temporal fascia was revealed. **l** The anastomotic nerves lie within the interfascial fat pad deep to the superficial layer of temporal fascia. **m** Temporalis muscle has been exposed following the retraction of deep layer of the temporal fascia. **n** The subfascial fat pad is located between the deep layer of the temporal fascia and the temporalis muscle. **o-p** The temporalis muscle periostium has been exposed deep to the temporalis muscle. *(Br: branch, Fasc: fascia, Front: frontal, Musc: muscle, Par: parietal, Subfasc: Subfascial, STA: superficial temporal artery, STL: superior temporal line, Temp: temporal)*

**Fig. 2:**
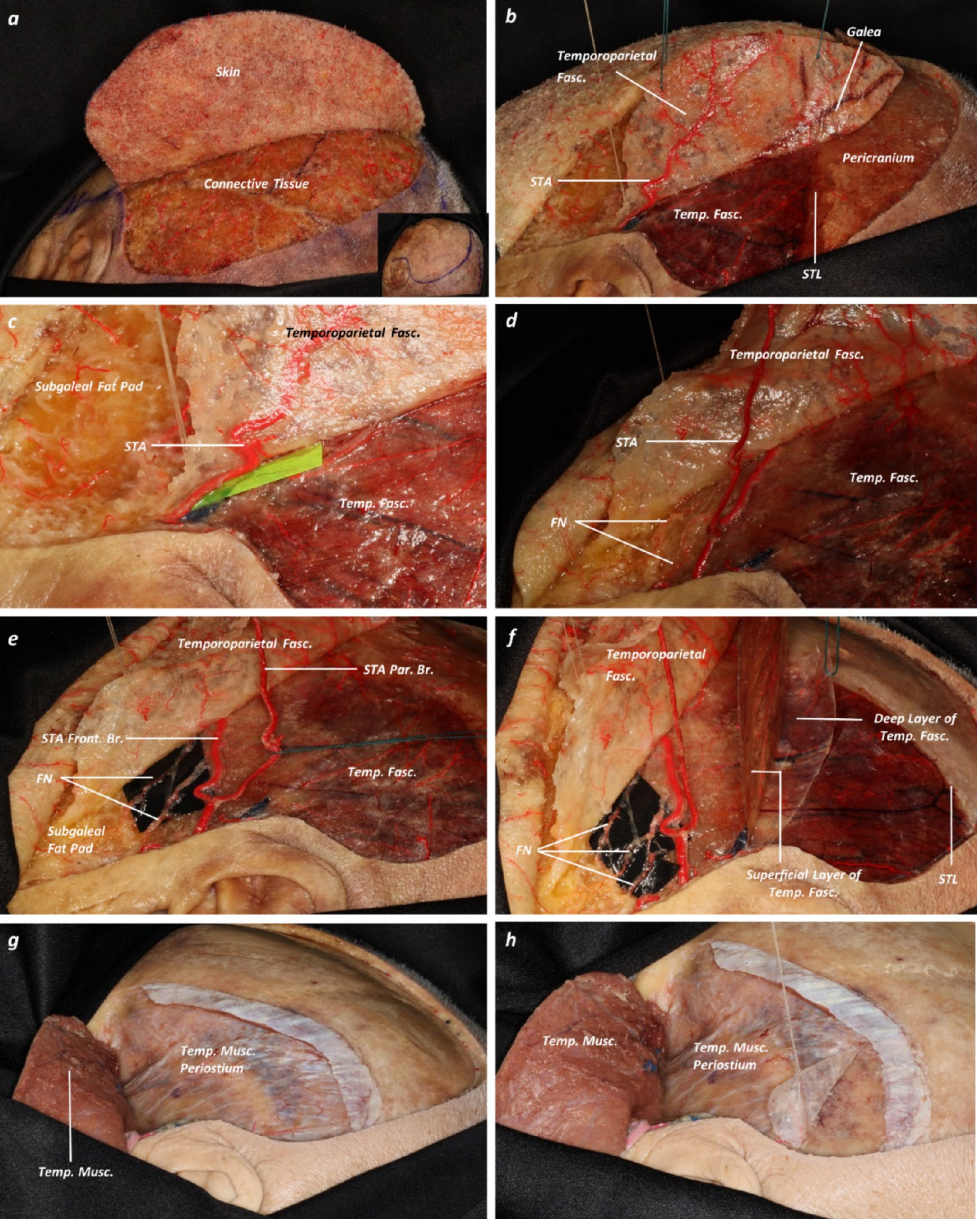
Layer by layer SCALP dissection after left-sided frontoparietotemporal skin incision. **a** The S layer of SCALP was dissected and elevated, and the C layer was revealed. Branches originating from STA and occipital artery – the main arteries responsible for SCALP nutrition – and dense anastomoses between them are located the C layer. The galea layer is located deep in the C layer. *(The inset shows the skin incision line.).* **b** The C layer has been completely removed and the galea layer has been partially elevated to show the continuity of the galea layer and temporoparietal fascia lateral and inferior to the STL. STA runs into the galea and temporoparietal fascia. The loose areolar tissue is medial to the STL and situated deep in the galea. The subgaleal fat pad between the temporoparietal fascia and the temporal fascia has been exposed. **c** Enlarged view. STA traverses along the temporoparietal fascia. **d-e** Dissection of the temporoparietal fascia has been proceeded more anteriorly. The temporal branches of facial nerve are located within the subgaleal fat pad. Frontotemporal branches of the facial nerve lie anterior and deep to the STA. **f** The temporal fascia, which is the continuation of the pericranium medial to the STL, is separated into two layers: the superficial layer (superficial layer of the temporal fascia) and the deep layer (deep layer of the temporal fascia). **g-h** The temporalis muscle periostium is located deep to the temporalis muscle and lies over the calvarium. *(Fasc: fascia, FN: facial nerve, Front. Br: frontal branch, Loose Ar. T: loose areolar tissue, Musc: muscle, Par. Br: parietal branch, Pericr: pericranium, STA: superficial temporal artery, STL: superior temporal line, Temp: temporal)*

**Fig. 3:**
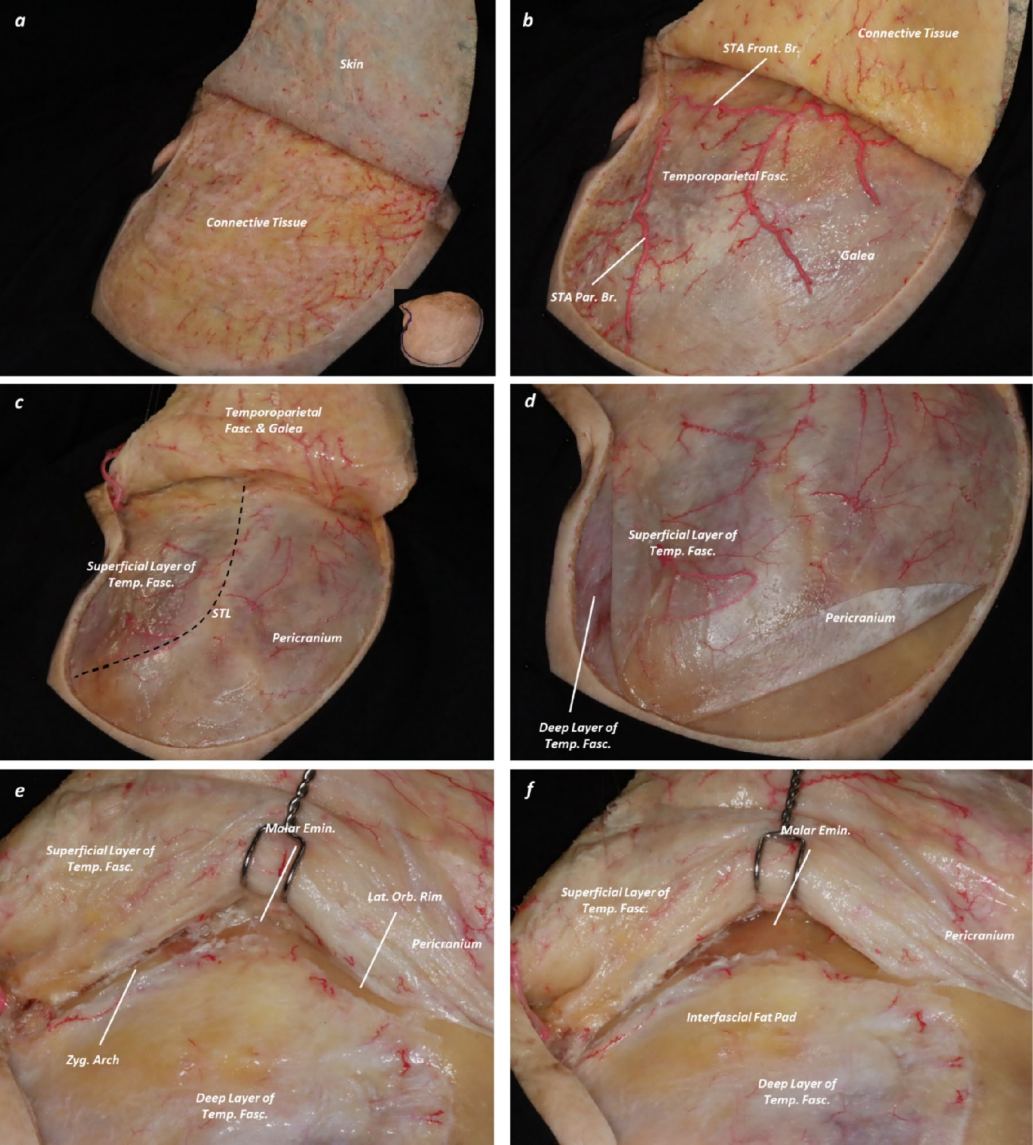

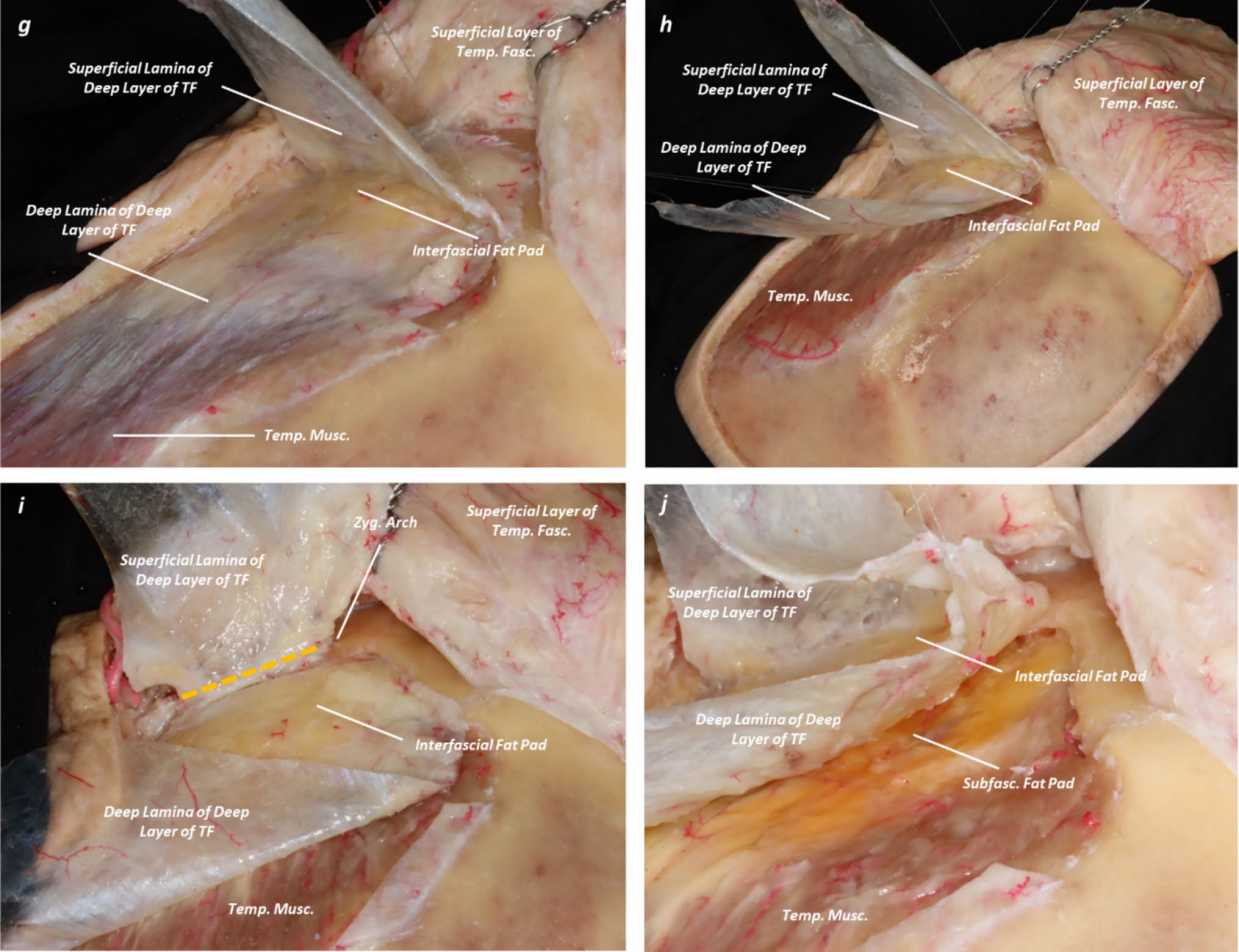
Anatomical relationships between the temporal fascia layers, the zygomatic arch and interfascial fat pad. **a** After a left-sided frontotemporoparietal skin incision, the skin has been elevated and the connective tissue has been exposed. *(The inset shows the skin incision.).* **b** Upon removal of the connective tissue, the temporoparietal fascia and its continuity – the galea – have been exposed. The main branches of the STA lie within the temporoparietal fascia. **c.** Following elevation of the temporoparietal fascia and galea, the pericranium and the temporal fascia have been displayed. The temporal fascia is continuous with the pericranium superomedial to the superior temporal line.* (Black dashed lines indicate STL.)* **d** The temporoparietal fascia has been elevated to expose the temporal fascia, which is separated into two layers—superficial and deep—with the superficial layer continuous with the pericranium and extending anteriorly to form the periorbita. **e-f** The superficial layer of the temporal fascia has been dissected from its deep layer, exposing the zygomatic arch, malar eminence and lateral orbital rim. The deep layer of the temporal fascia attaches to these structures. The reflection of the interfascial fat pad is visible on the surface of the deep layer of the temporal fascia. The superficial layer of the temporal fascia extended inferiorly over the zygomatic arch and malar eminence without firm adhesion, which may clearly aid orbital osteotomies during cranio-orbito-zygomatic approach (COZA). **g-h-i** The deep layer of the temporal fascia consisted of two laminae, lateral and medial. The lateral lamina adhered to the lateral surface of the zygomatic arch, and the medial lamina adhered to its medial surface. The medial lamina coursed over the temporalis muscle. The interfascial fat pad lie between the lateral and medial laminae at the level of the zygomatic arch.* (The yellow dashed line indicates the location of the zygomatic arch.)* **j** The lateral and medial laminae of the deep layer of the temporal fascia merged on the superior surface of the zygomatic arch. (*Emin: eminence, Fasc: fascia, Front. Br: frontal branch, Lat: lateral, Musc: muscle, Orb: orbital, Par. Br: parietal branch, Subfasc: subfascial, Temp: temporal, TF: temporal fascia, Zyg: zygomatic)*

**Fig. 4:**
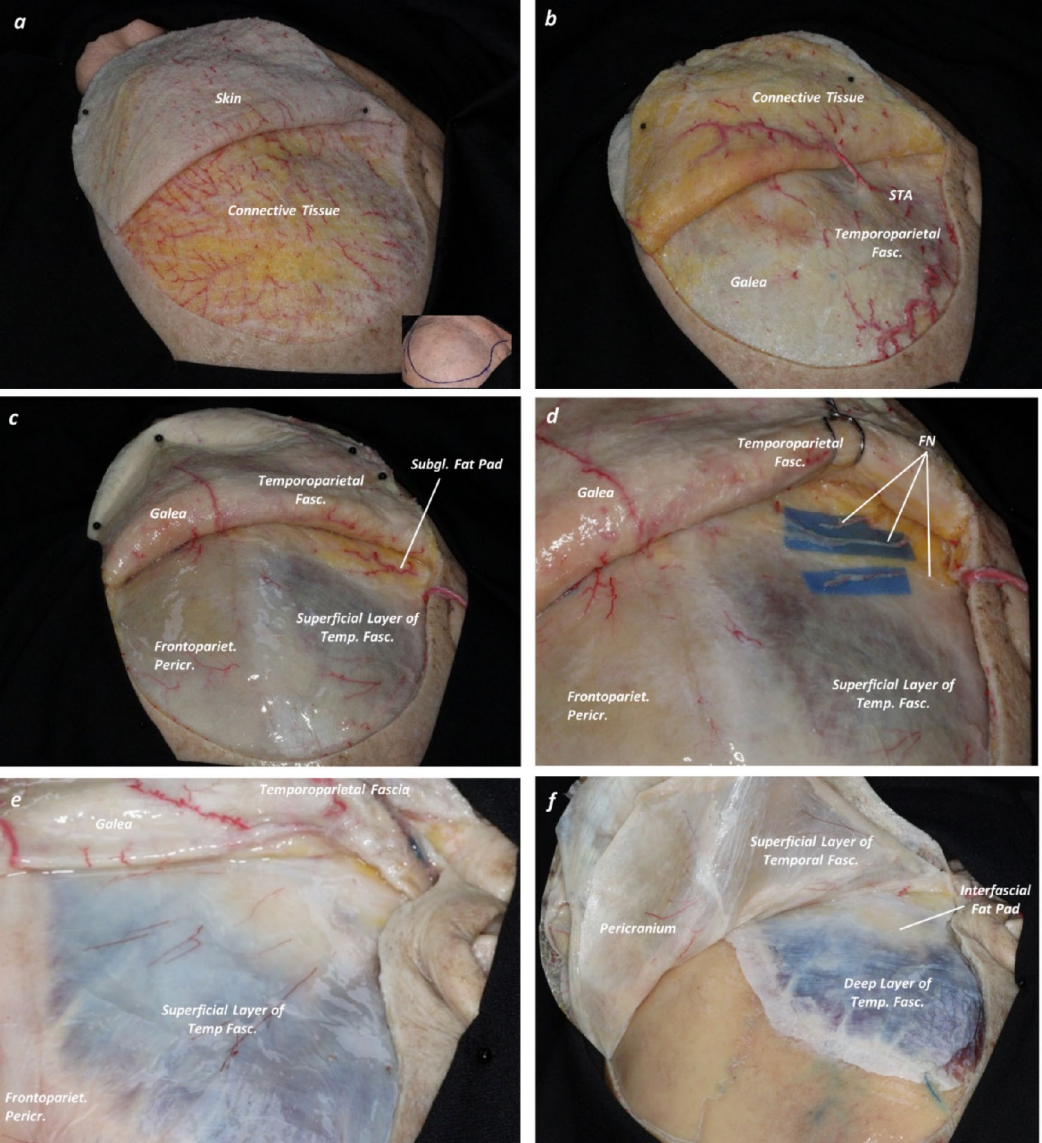

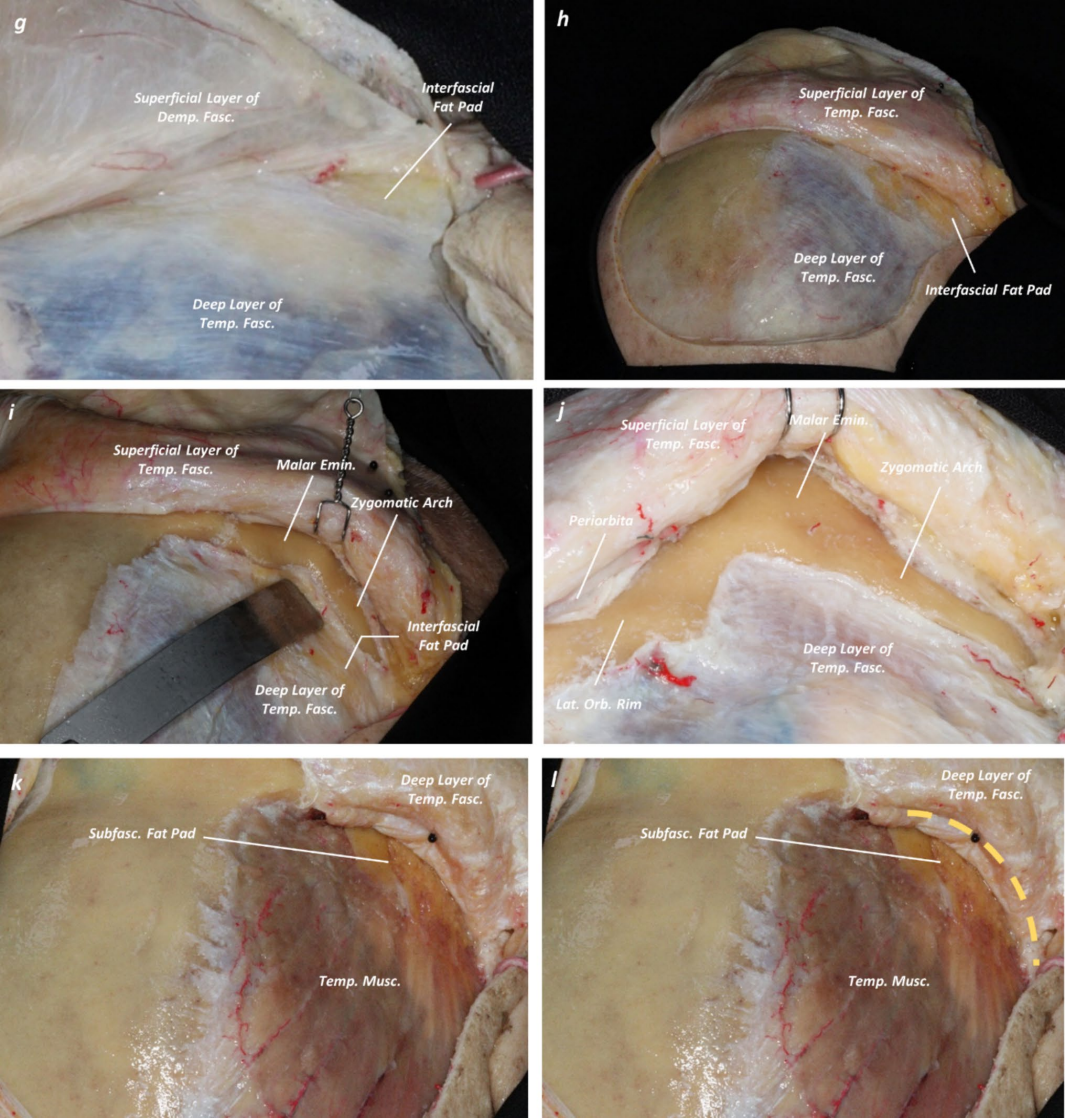
Anatomical relationships between zygomatic arch and temporal fascia** a** With the head rotated to the contralateral side, a right-sided frontotemporal skin incision has been performed to expose the connective tissue and the distal branches of STA within it. *(The inset shows the skin incision*.*).* **b** After the removal of the connective tissue, the galea and its inferolateral continuation – the temporoparietal fascia – have been exposed. The proximal STA courses within the temporoparietal fascia and becomes superficial toward the connective tissue distally.** c** By removing the galea and temporoparietal fascia, the pericranium and the superficial layer of the temporal fascia have been exposed. The subgaleal fat pad is located between the temporoparietal fascia and the superficial layer of the temporal fascia.** d** The frontotemporal branches of the facial nerve are located within the subgaleal fat pad. **e-h** The superficial layer of the temporal fascia together with the frontoparietal pericranium have been elevated to expose the deep layer of the temporal fascia. The reflection of the interfascial fat pad located in between the laminae of the deep layer of the temporal fascia can be appreciated. **i** The superficial layer of the temporal fascia continued downward without adhering to the zygomatic arch and this layer was dissected from the deep layer of the temporal fascia. **j** After retracting the superficial layer of the temporal fascia downward, the deep layer was found to adhere to the lateral surfaces of the zygomatic arch, malar eminence, and lateral orbital rim. The superficial layer of the temporal fascia extended inferiorly over the zygomatic arch without firm adhesion and maintained continuity with the periorbita anteriorly, which may facilitate orbital osteotomies during cranio-orbito-zygomatic approach (COZA). **k** Removal of the deep layer of the temporal fascia exposed the temporalis muscle and the subfascial fat pad situated between them. The deep layer adhered to the lateral and medial borders of the zygomatic arch and merged along its superior border. **l** The yellow dashed line indicates the location of the zygomatic arch. The subfascial fat pad is located deep to the zygomatic arch. Deep layer adhered to the lateral and medial borders of the zygomatic arch and merged along its superior border. *(Emin: eminence, Fasc: fascia, FN: facial nerve, Interfasc: interfascial, Pericr: pericranium, Subfasc: subfascial, Subgl: subgaleal, STA: superficial temporal artery, Temp: temporal)*

After frontotemporoparietal skin incision with head in 30–45 degrees rotation to contralateral side, the skin layer (S layer) was dissected from the connective tissue (C layer) just below it (Figs. 1a, 2a and 3a). The branches of the superficial temporal artery (STA) were identified in C layer (Figs. 1a, 2a, 3a-b, and 4a-b). Next, the C layer was elevated and the galea (A layer, aponeurosis) was revealed underneath the connective tissue (Figs. 1b, 2b, 3b and 4b) Lateral and inferior of the STL, galea was continuous with the TPF (Figs. 1b-c, 2b, 3b and 4b) (Table 1). STA was located in the TPF at the level of the tragus and its branches subsequently became superficial towards the connective tissue as they move medially (Figs. 1c, 2b-c, 3b and 4b). As loose areolar tissue (L layer) formed a good dissection plane between the galea and pericranium, galea layer and loose areolar tissue were dissected together.

**Table 1:** Continuity of the layers in the superomedial and inferolateral to STL and the structures they contain. The terminology and nomenclature of the fascial layers used in this study were adapted from Davidge et al. [12] and Krayenbühl et al. [20]

Layer superior (medial) to STL	Continuing Layer Inferior (lateral) to STL	The Structures It Contains
Galea	Temporoparietal fascia (superficial temporal fascia)	STA
Loose areolar tissue	Subgaleal fat pad (suprafacial fat pad)	Temporal branches of the facial nerve
Pericranium	**Temporal fascia (deep temporal fascia):** It consists of two layers ***Superficial layer of temporal fascia:*** It continues with the periorbita ***Deep layer of temporal fascia*:** It consists of two layers and the interfascial fat pad is located between these two: ***Lateral lamina:*** Attaches to the lateral surface of the zygomatic arch ***Medial lamina:*** Attaches to the medial surface of the zygomatic arch	Sensory anastomotic branches between the facial nerve and the trigeminal nerve Interfascial fat pad

*STA* superficial temporal artery, *STL* superior temporal line

TPF was then elevated anteroinferiorly from the underlying TF, which was divided into two layers—superficial and deep—with the superficial layer continuous with the pericranium and extending anteriorly to form the periorbita (Figs. 1d, 2b, 3c-d and 4c). The subgaleal (suprafacial) fat pad was identified between the TPF and TF and the fronto-temporal branches of the facial nerve were encountered at this subgaleal level (Figs. 1e-g, 2c-e, 4c-d) (Table 2) (Figs. 1f, 2c-e, 4c-d) (Table 1).

**Table 2:** Localization of the three different fat pads and the structures they contain

Fat Pad	Localization	The Structures It Contains
Subgaleal (Suprafacial)	Between temporoparietal fascia and superficial layer of the temporal fascia	Frontotemporal branches of the facial nerve
Interfascial (Superficial)	Between lateral and medial laminas of the deep layer of temporal fascia	Sensory anastomotic branches between the facial nerve and the trigeminal nerve
Subfascial (Deep)	Between deep lamina of the deep layer of temporal fascia and temporalis muscle	Small anastomotic branches between the STA and the maxillary artery

*STA:* superficial temporal artery

At the level of the STL, the TF was observed to split into superficial and deep layers (Figs. 1h–l, 2f, 3c–f, 4e–f). Our dissections demonstrated that the pericranium (P layer, periosteum), located beneath the galea, continues as the superficial layer of TF lateral and inferior to the STL (Figs. 1b and d, 2b, 3c and 4c) (Table 1). The superficial layer of the TF extended inferiorly over the zygomatic arch and ME without firm adhesion and maintained continuity with the periorbita anteriorly. This anatomical arrangement created a distinct interfascial plane over the zygomatic arch and ME during COZA (Fig. 3e–f, 4g–j).

The deep layer of TF was further composed of two laminae – lateral and medial –with an interfascial fat pad situated between them (Fig. 3g–j) (Tables 1 and 2). The lateral and medial laminae attached to the lateral and medial surfaces of the zygomatic arch, respectively, and merged at the superior margin. (Fig. 3g–j, 4k–l). The reflection of interfascial fat pad was also visible on the surface of the deep layer of the TF (Figs. 1k, 3f, 4f–i). An important anatomical observation in our dissections was the presence of anastomotic branches of the facial nerve traversing the interfascial fat pad between the lateral and medial laminae (Fig. 1L).

The final step of dissection involved removing deep layer of the TF, which uncovered the subfascial fat pad lying between the temporalis muscle and the fascia (Figs. 1m–n, 3j, 4k–l) (Table 2). After dissecting the temporalis muscle from the STL, the temporalis muscle periostium was revealed between the muscle and calvarium (Fig. 1o–p, 2g–h).

### SCALP dissection in relation to the facial nerve

**Fig. 5:**
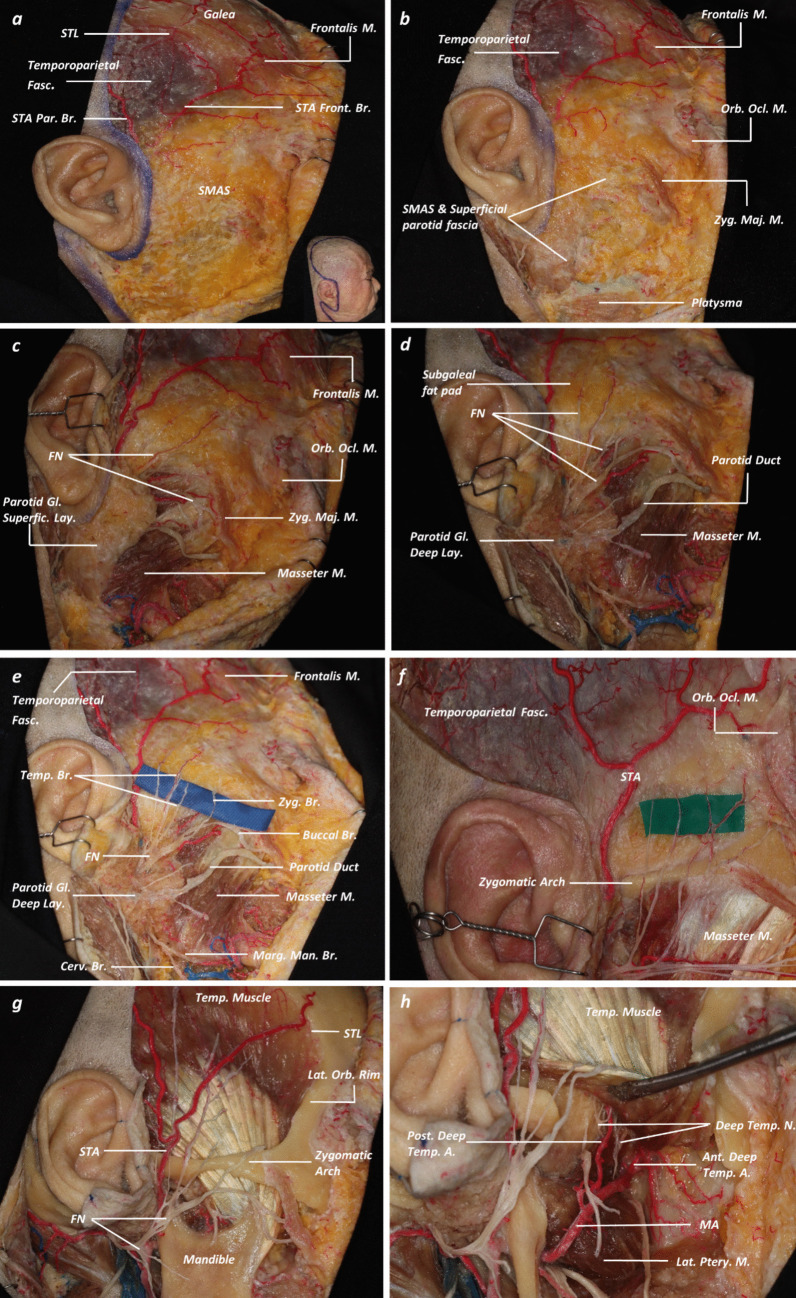
Right side superficial to deep facial dissection showing the relationship between the layers of the face, facial nerve branches, temporoparietal fascia and temporalis muscle. **a** The inset demarcates the skin incision. The facial region is composed of four distinct layers: the skin, subcutaneous fatty tissue, the superficial musculoaponeurotic system (SMAS) and the parotid–masseteric fascia. Following removal of the skin and subcutaneous fat tissue, the galea has been exposed above the zygomatic arch. The frontalis muscle and the superficial temporal artery (STA) are located within the galea. Inferior to the superior temporal line (STL), the temporoparietal fascia continuous with the galea. The SMAS has been revealed after removal of skin and subcutaneous tissue inferior to the zygomatic arch. The SMAS and the temporoparietal fascia lie at the same anatomical level. **b** The SMAS is continuous with the superficial fascia of the parotid gland and the platysma in the neck. The SMAS and temporoparietal fascia are positioned at the same level. The frontalis muscle is located within the galea, whereas the orbicularis oculi and zygomaticus major muscles are situated within the SMAS. **c** After removing the SMAS along with the continuous superficial fascia of the parotid gland, the masseter muscle and the superficial layer of the parotid gland were exposed. After emerging from the stylomastoid foramen, the branches of the facial nerve course between the superficial and deep lobes of the parotid gland. The frontotemporal branches of the facial nerve course within the subgaleal fat pad and innervate the orbicularis oculi, frontalis and corrugator supercilii muscles. **d–e** The SMAS and the superficial layer of the parotid gland have been removed, exposing the masseter muscle, the deep layer of the parotid gland and the facial nerve branches located laterally. After exiting the stylomastoid foramen, the facial nerve runs in between the superficial and deep layers of the parotid gland and divides into distinct branches: temporal, zygomatic, buccal, marginal mandibular, cervical. **f** The SMAS and parotid gland have been removed. The facial nerve branches course superficially to the zygomatic arch and at the level of the arch, they are positioned anteriorly and deep relative to the STA. While the STA is situated within the temporoparietal fascia, the facial nerve branches reside within the subgaleal fat pad. **g** The mandible, zygomatic arch, malar eminence and temporalis muscle have been exposed after removal of the SMAS, masseter muscle, temporoparietal fascia, temporal fascia and fatty planes. The temporalis muscle extends between the coronoid process of the mandible and the STL. The frontotemporal branches of facial nerve run over the zygomatic arch and lie anterior and deep to the STA at that level. **h** The mandible and zygomatic arch have been removed, the temporalis muscle retracted superolaterally to expose the infratemporal fossa. The anterior and posterior deep temporal arteries, originating from the maxillary artery in the infratemporal fossa, supply the temporalis muscle from its medial side. The deep temporal nerves, originating from the anterior division of the mandibular nerve, innervate the temporalis muscle. *(A: artery, Ant: anterior, Br: branch, Buc: buccal, Cerv: cervical, Fasc: fascia, FN: facial nerve, Front: frontal, Gl: gland, Lay: layer, Lat: lateral, M: muscle, MA: maxillary artery, Maj: major, Mand: mandibular, Marg: marginal, N: nerve, Par: parietal, Ocl: oculi, Orb: orbicularis, Pericr: pericranium, Post: posterior, Ptery: pterygoid, SMAS: superficial musculoaponeurotic system, Superfic: superficial, STA: superficial temporal artery, STL: superior temporal line, Temp: temporal, Zyg: zygomatic)*

**Fig. 6:**
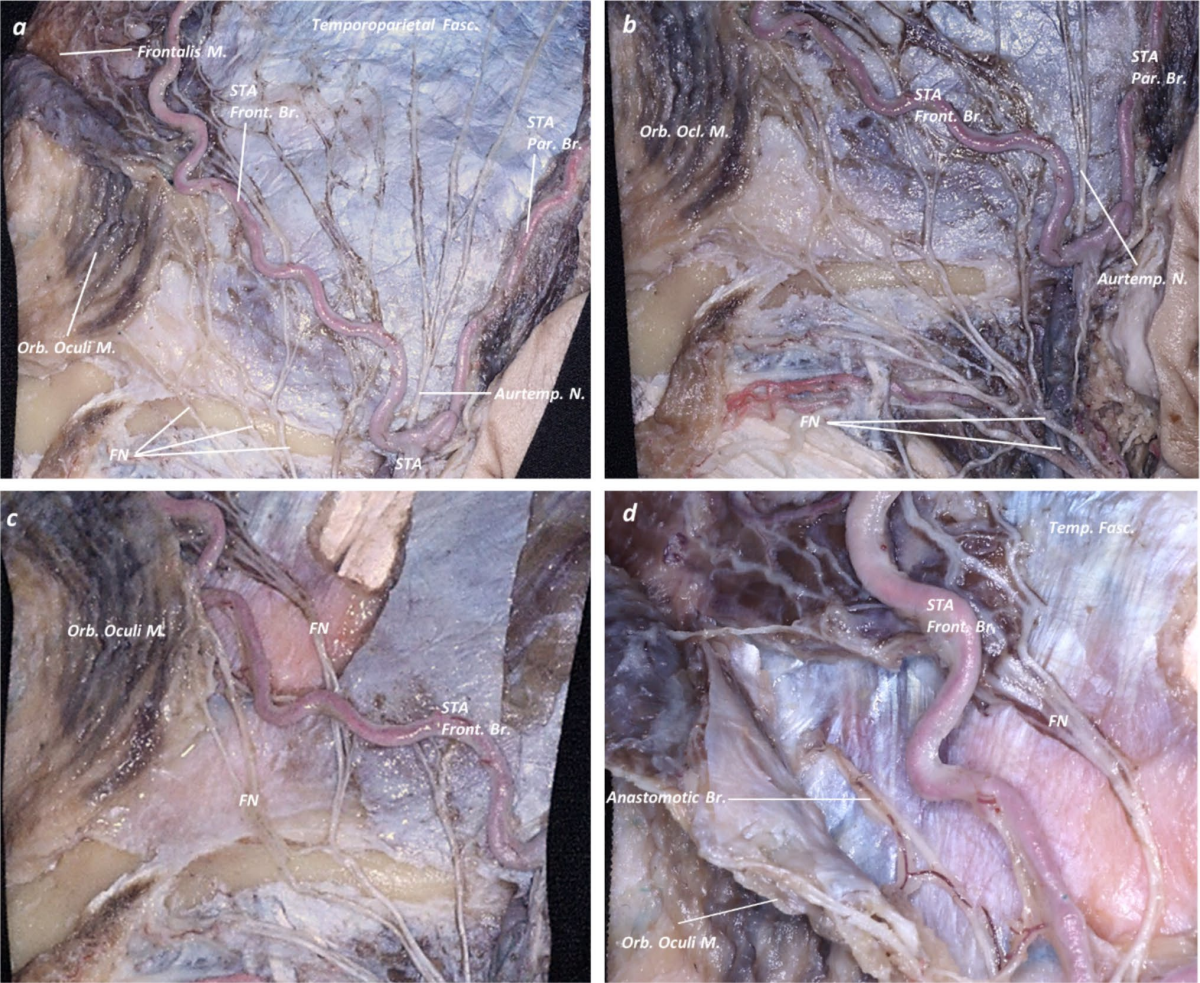
Lateral to medial facial dissection following a preauricular skin incision to display the course of the frontotemporal branches of the facial nerve. **a–b** After the skin and connective tissue were removed, the galea and its continuation, the temporoparietal fascia, were clearly exposed. By removal of the subgaleal fat pad, the course of the zygomatic arch and frontotemporal branches of the facial nerve have been revealed. The frontotemporal branches of the facial nerve pass towards the frontalis muscle and orbicularis oculi muscles. The frontalis and orbicularis oculi muscles were identified at the level of the galea. The STA course along the temporoparietal fascia. At the level of the zygomatic arch, the facial nerve branches are located anterior to the STA. The auriculotemporal nerve lies posterior to the frontotemporal branches of facial nerve. **c** The frontotemporal branches of the facial nerve course towards the orbicularis oculi and frontalis muscles. **d** After the orbicularis oculi muscle was elevated along with the superficial layer of TF, the anastomotic nerves are located at the level of deep layer of TF have been exposed. *(Aurtemp: auriculotemporal, DTF: deep temporal fascia, Fasc: fascia, Fr: frontal, FN: temporal branch of facial nerve, M: muscle, N: nerve, Orb: orbicularis, Par: parietal STA: superficial temporal artery)*

Following a preauricular incision extending to neck, skin and subcutaneous fat tissue were removed, exposing the galea, the TPF and superficial musculoaponeurotic system (SMAS). TPF and SMAS were at the same level (Fig. 5a–b). Frontotemporal branches of the facial nerve traversed superficial to the zygomatic arch. Facial nerve branches coursed anterior and deep to the STA at the level of the zygomatic arch and coursed within the subgaleal fat pad above the arch to ascend anteriorly towards the orbicularis oculi and frontalis muscles (Fig. 5c–f, 6a–c). Anastomotic sensory branches were revealed deep to the subgaleal fat pad (Fig. 6d). The anterior and posterior deep temporal arteries and deep temporal nerves supply and innervate the temporalis muscle, respectively (Fig. 5g-h).

### Preservation of facial nerve branches: interfascial and subfascial dissections

#### Interfascial dissection

After frontotemporal skin incision, skin, C layer, galea and the TPF were dissected at the level of loose areolar tissue. The TPF and galea were dissected together and harvested as a vascularized pedicled TPF-galeal flap (Fig. 1a-j). The pericranium was dissected over the calvarium separataly to obtain pericranial flap (Fig. 1i-m). At the level of the STL, following an incision in the superficial layer of the TF, this layer was elevated together with the subgaleal fat pad, thereby preserving the facial nerve branches. When the superficial layer of the TF was separated from its deep layer and followed inferiorly, zygomatic arch and the ME were reached. (Fig. 3d–f, 4f–i).

#### Subfascial dissection

Frontotemporoparietal skin incision was performed to elevate skin and the C layer and galea and the TPF were then dissected together, revealing pericranium and the TF (Fig. 3a–d). The TF and pericranium, can be elevated together and harvested for skull base reconstruction (Fig. 3a–d, 4f). During the subfascial dissection technique, all layers of the TF were incised down to the level of the temporalis muscle to preserve the facial nerve branches. An additional incision in the deep layer of the TF was required to expose the zygomatic arch (Fig. 4k–l).

## Discussıon

Temporalis muscle periostium, located on the calvarial side of temporalis muscle, is a continuation of frontoparietal pericranium [19, 20]. Accordingly, frontoparietal pericranium can be considered continuous with three distinct layers inferolateral to the STL: the superficial and deep layers of the TF and temporalis muscle periostium (Fig. 1o–p, 2g–h).

Terminology of the SCALP layers remains inconsistent in the literature. Different terms have been used for the fascial layers overlying the temporalis muscle, including temporal fascia, deep temporal fascia and superficial or deep layers of the temporal fascia. Similarly, temporoparietal fascia has also been referred to as superficial temporal fascia in several anatomical studies. In the present study, the terminology proposed by Davidge et al. was adopted for consistency. Equivalent terminology used in the literature is summarized in Table 1 [5, 12, 20, 26].

### Comparison of dissection techniques for preservation of facial nerve branches

Whether the TPF is truly continuous with the SMAS remains a matter of debate; nonetheless, the TPF follows the same anatomical plane as the SMAS above the zygomatic arch (Fig. 5 and 6) [1, 2, 8, 17, 20, 22]. Frontotemporal branches of the facial nerve traverse the subgaleal fat pad and positioned deep in the TPF above the zygomatic arch; whereas below the arch, the nerve branches lie deep in the SMAS (Fig. 5 and 6) [26]. Injury to these branches may result in frontalis muscle dysfunction [1, 11, 37].

Three distinct dissection techniques have been suggested for preserving the facial nerve branches during pterional craniotomy and COZA: submuscular method, interfascial technique and subfascial method [11, 37]. Although the submuscular technique minimizes the risk of facial nerve injury, it is less optimal for COZA due to limited exposure of the zygomatic arch, the lateral orbital wall and ME up to the level of zygomaticofacial foramen [11, 37]. Consequently, interfascial and subfascial techniques are more commonly employed when adequate retraction are required [11, 37].

This study demonstrates that the TF splits into superficial and deep layers at the level of the STL. Our dissections underscore the critical role of the superficial layer of TF in preserving facial nerve branches during COZA, highlighting several key anatomical factors. The superficial layer of TF extends inferiorly over the zygomatic arch without firm adhesion and maintains anterior continuity with the periorbita. These findings suggest that following the superficial layer of the TF inferiorly during interfascial dissection provides a natural plane between the superficial and deep layers that preserves the facial nerve branches while facilitating exposure of the ME, lateral orbital wall and IOF during COZA [6, 10, 32, 33].

In our dissections, the deep layer of TF was further separated into two laminae – lateral and medial –with an interfascial fat pad situated between them (Fig. 3g–j). These two laminae typically merge at the superior edge of the zygomatic arch and reflection of the interfascial fat pad can be observed consistently. Anastomotic branches of the facial nerve were identified traversing this fat pad between these two laminae. Interfascial fat pad can also be helpful in maintaining the correct dissection plane.

Núñez et al. supported the reliability of the interfascial dissection technique by demonstrating that intraoperative stimulation of the trigeminofacial anastomotic branch – located between the layers of the TF – elicited no motor response from the facial nerve, and that sacrificing this branch did not affect frontalis muscle function [23]. Spiriev et al. reported that no frontotemporal facial nerve branches were identified within the interfascial fat pad and concluded that interfascial dissection could be performed without risk of frontalis muscle palsy [29].

Subfascial dissection requires an incision along the superior edge of the zygomatic arch through the adherent superficial layer of the TF, which may place the subgaleal and interfascial branches at risk. In contrast, interfascial dissection allows direct identification of the fascial layers at the level of the zygoma, where the layers separate naturally (Fig. 3 and 4).

At the level of the zygomatic arch, facial nerve branches lie anterior to the STA (Fig. 5e–g). Accordingly, fascia incisions made posterior to the STA at this level are unlikely to cause injury to the facial nerve [18, 33]. Campero et al. described “facial-zygomatic triangle”, as a landmark to preserve both the facial nerve and STA during dissection around the zygomatic arch [7]. Poblete et al. reported that facial nerve branches course within the subaponeurotic plane (loose areolar tissue) and recommended 'subpericranial–interfascial' or 'subpericranial–subfascial' dissection technique to reduce nerve injury [10, 26]. They also noted that galeal incisions within 4 cm of the lateral orbital rim may endanger the frontotemporal branches [26]. Salas et al. described an area near the zygomatic arch where the galea–TPF complex becomes adherent to the superficial layer of the TF. They emphasized that subaponeurotic dissection in this region may place the facial nerve branches at risk and therefore recommended interfascial dissection to reach the zygomatic arch. In our dissections, following the superficial layer of the TF inferiorly provided a clear interfascial plane over the zygomatic arch [28].

STL represents a key anatomical junction where the galea, pericranium, fasciae and muscle converge in the frontotemporal region, creating a suitable fascial detachment plane just above the temporalis muscle. To separate the superficial and deep layers of the TF, a TF incision can be made at the level of the STL so that the interfascial surgical plane is effectively identified [30]. Medial to STL, the pericranium and the superficial layer of TF should be dissected as a single layer.

### Vascularized flaps and interfascial dissection

Vascularized temporoparietal fascia flaps and pericranial flaps are commonly used for reconstruction and dural repair [14, 35, 38]. Elevation of these continuous layers across the STL allows creation of larger vascularized flaps. During this step, preservation of the arterial supply and the facial nerve branches coursing between the layers is essential. Loose areolar tissue provides a useful plane for pericranial flap elevation [2, 12, 13, 26, 31, 35]. Pericranium may also be elevated together with the continuation of the temporal fascia to obtain a larger vascularized flap [37]. During TPF flap harvesting, interfascial dissection along the superficial layer of the temporal fascia may help preserve the facial nerve branches within the subgaleal plane.

### Limitations

Use of formalin-fixed specimens may alter tissue properties and ease of fascial separation compared with live surgical conditions. This study was descriptive in nature; no quantitative morphometric measurements were performed. No clinical series were included to correlate these anatomical findings with surgical outcomes. However, the step-by-step anatomical observations presented here can help guide the dissection process during COZA.

## Conclusions

The superficial layer of TF extends inferiorly over the zygomatic arch and ME without firm adhesion and maintains anterior continuity with the periorbita. The plane between the superficial and deep layers of the TF provides a practical interfascial dissection corridor that allows tissue separation while preserving the frontotemporal branches of the facial nerve during COZA. Recognition of this anatomical relationship may facilitate exposure of ME, lateral orbital wall and IOF and assist orbital osteotomies. Awareness of the interfascial fat pad between the deep layers of the TF and their attachments along the zygoma may prove helpful during surgery.

## Data Availability

No datasets were generated or analysed during the current study.

## Abbreviations

COZA: Cranio-orbito-zygomatic approach
IOF: Inferior orbital fissure
ME: Malar eminence
STA: Superficial temporal artery
STL: Superior temporal line
TF: Temporal fascia
TPF: Temporoparietal fascia
